# Narrative Review: Advancing Dysbiosis Treatment in Onco-Hematology with Microbiome-Based Therapeutic Approach

**DOI:** 10.3390/microorganisms12112256

**Published:** 2024-11-07

**Authors:** Salomé Biennier, Mathieu Fontaine, Aurore Duquenoy, Carole Schwintner, Joël Doré, Nathalie Corvaia

**Affiliations:** 1 MaaT Pharma, 69007 Lyon, France; sbiennier@maat-pharma.com (S.B.); aduquenoy@maat-pharma.com (A.D.); cschwintner@maat-pharma.com (C.S.); ncorvaia@maat-pharma.com (N.C.); 2 Université Paris-Saclay, INRAE, MetaGenoPolis, AgroParisTech, MICALIS, 78350 Jouy-en-Josas, France; joel.dore@inrae.fr

**Keywords:** dysbiosis, GvHD, onco-hematology, donor-derived products, full ecosystem

## Abstract

This review explores the complex relationship between gut dysbiosis and hematological malignancies, focusing on graft-versus-host disease (GvHD) in allogeneic hematopoietic stem cell transplantation (allo-HSCT) recipients. We discuss how alterations in microbial diversity and composition can influence disease development, progression, and treatment outcomes in blood cancers. The mechanisms by which the gut microbiota impacts these conditions are examined, including modulation of immune responses, production of metabolites, and effects on intestinal barrier function. Recent advances in microbiome-based therapies for treating and preventing GvHD are highlighted, with emphasis on full ecosystem standardized donor-derived products. Overall, this review underscores the growing importance of microbiome research in hematology–oncology and its potential to complement existing treatments and improve outcomes for thousands of patients worldwide.

## 1. Introduction

The human gut microbiome has emerged as a critical factor in host health, playing pivotal roles in metabolism, nutrition, physiology, and immune functions. This complex ecosystem, comprising trillions of microorganisms including more than 1000 bacterial species, viruses, fungi, and protozoa, has been the subject of extensive research in recent years, revealing its significant impact on human biology. The symbiotic relationship between the host and gut microbes provides numerous benefits that extend far beyond the gastrointestinal tract, influencing various aspects of human physiology and health.

A disruption in the composition and function of the gut microbiome, known as dysbiosis, has been associated with a wide range of diseases, including inflammatory bowel disease (IBD) [1], irritable bowel syndrome (IBS) [2], obesity [3], type 2 diabetes [4], cardiovascular diseases [5], neurodegenerative disorders [6], neuropsychiatric disorders, troubles of neuro-development [6,7], and cancers [8]. Mechanisms underlying dysbiosis and the impact on diseases are complex and multifaceted, involving alterations in barrier integrity [9,10], metabolic functions [11], and immune regulation [12,13]. In recent years, multiple studies have shown the crucial role of inflammation and diet on the intestinal barrier permeability and its direct impact on disease development. Indeed, the intestinal mucosa encounters several exogenous antigens and pro-inflammatory molecules, including dietary antigens, food-borne pathogens, and commensal bacterial present in the gut lumen. Consequently, in the physiological state, the gut acts as a barrier tissue composed of a monolayer of intestinal epithelial cells linked by tight junctions, which helps in maintaining biological homeostasis [14]. Inflammatory conditions often driven by an imbalance in the gut microbiota composition can increase intestinal permeability, enabling pro-inflammatory molecules to enter systemic circulation, which directly contributes to the chronic inflammation of the gut. This is particularly evident in metabolic disorders such as obesity, inflammatory bowel disease (IBD), and cardiovascular diseases [15], where increase gut permeability was demonstrated to exacerbate systemic inflammation [16,17].

Furthermore, this disruption of the gut intestinal barrier integrity and this inflammation state is a critical factor in tumorigenesis [18]. It has been demonstrated over the years that dysbiosis is linked with cancers development and progression, including colorectal cancer (CRC), through mechanisms such as the accumulation of microbial toxins and metabolites known to modulate immune responses [19,20]. Certain pathogenic bacteria, especially *Fusobacterium nucleatum* and *Bacteroides fragilis*, have been associated with CRC due to their ability to disrupt gut integrity and activated inflammatory pathways [20,21]. Additionally, dysbiosis can influence the tumor microenvironment and affect the efficacy of anticancer therapies by altering drug metabolism and immune responses [22,23].

As our understanding of the gut microbiome continues to grow, this review aims to provide a comprehensive overview of the current state of knowledge regarding the gut microbiome dysbiosis, its implications in cancer, and the emerging field of microbiome-based therapies, with a particular focus on applications of fecal microbiome-based therapy in hematological malignancies.

### 1.1. Gut Microbiome Composition and Development

The gut microbiome is dominated by four main phyla: Bacillota [Firmicutes], Bacteroidota [Bacteroidetes], Actinomycetota [Actinobacteria], Pseudomonadota [Proteobacteria] [24]. In total, 90% of the bacteria in the intestinal microbiota are from the Bacteroidetes or Firmicutes phyla. In fact, there is a ratio, the Firmicutes/Bacteroidetes ratio, which considers a microbiota to be “healthy” when it is equivalent to 1:1 [25,26]. Among these, the *Bacteroides* genus is typically the most abundant in the adult gut microbiome, playing a crucial role in the breakdown of complex carbohydrates and the production of short-chain fatty acids (SCFAs). These latter metabolites, especially butyrate, were found to have an impact on gut barrier integrity [27], possess anti-inflammatory effects [28], serve immunomodulation functions [29], influence metabolism [9], and aid in cancer prevention [30]. Other common and abundant genera include *Faecalibacterium*, *Roseburia*, and *Bifidobacterium*, each contributing uniquely to gut health and metabolic functions [31]. In addition to bacteria, the gut microbiome also harbors archaea, primarily *Methanobrevibacter smithii*, eukaryotes such as yeasts, and a diverse array of viruses, predominantly bacteriophages [32].

The intestinal microbiota is composed of different compartments with distinct functions. Its composition varies according to the part of the digestive tract in which it evolves. The digestive tract begins in the mouth, followed by the esophagus, the stomach, the small intestine (duodenum, jejunum, and ileum), the large intestine (ascending, transverse, and descending colon), and ends with the rectum. Recent studies have demonstrated that the oral microbiota plays a crucial role in the initial degradation of dietary compounds, including phenolic compounds, directly influencing the production of essential gut metabolites such as the SCFA. Furthermore, Elghannman et al. [33] highlighted the potential link between oral microbiota dysbiosis and gastrointestinal diseases, including colorectal cancer. For instance, *Fusobacterium nucleatum* and *Porphyronomas gingivalis* have been found to be enriched in patients with colorectal cancer, activating pro-inflammatory pathways and thus promoting colorectal tumorigenesis. It is important to note that these different parts of human digestive tract have their own physiological parameters with different environments of pH, oxygen saturation, and tension, as well as the presence of substrates, enzymes, and bile acids conditioning bacterial growth and thus bacterial composition. Additionally, factors such as diet, age, genetics, and environmental exposures contribute to this variability [12].

The establishment and maturation of the gut microbiome is a dynamic process that begins at birth and continues throughout an individual’s lifetime. The mode of delivery plays a crucial role in initial colonization, with vaginally delivered infants acquiring microbes resembling their mother’s vaginal microbiota, while those born via cesarean section are initially colonized by skin and environmental microbes [34]. Indeed, vaginally delivered infants acquire microbes resembling their mother’s vaginal microbiota, such as Lactobacillus, Prevotella, and Sneathais species, while those born via cesarean section are initially colonized by skin bacteria like Staphylococcus, Corynebacterium and Propionibacterium species. This developing microbiome plays a crucial role in educating and shaping the immune system. For instance, specific microbial species like segmented filamentous bacteria promote the development of Th17 cells, while certain commensal bacteria induce regulatory T cells (Tregs), promoting immune tolerance [35]. These initial differences can persist over the first year of life, with CS-born infants typically lacking Bacteroides species until 6–18 months of age and showing delayed acquisition of beneficial bacteria like Bifidobacterium [36,37]. The altered gut microbiota composition in CS-born infants may lead to prolonged postnatal immunological immaturity, affecting the development of gut-associated lymphoid tissues and the balance of Th1/Th2 immune responses [38]. Hence, this initial colonization sets the stage for the subsequent development of the microbiome, which undergoes rapid changes in composition and diversity during the first three years of life. In addition, factors such as breastfeeding, introduction of solid foods, antibiotic exposure, and their environment, contribute to shaping the developing microbiome. This early-life period is critical for the development of the immune system and metabolic programming, with potential long-term consequences for health and disease susceptibility [39]. Studies have shown that antibiotic exposure in infancy is associated with altered microbiome composition in later childhood and adulthood, potentially increasing the risk of developing asthma, allergies, and IBS [40]. Moreover, a large longitudinal study of 100 newborn infants found that microbial dysbiosis during the first 3 months of life was associated with increased circulating endothelial cells, activation of T cell populations, and higher levels of pancreatic digestive enzymes in blood samples at 3 months [40]. Furthermore, the microbiome also influences metabolic programming, with early-life microbiome composition being associated with obesity risk in later childhood. These complex interactions underscore the importance of the early-life period in establishing a healthy, diverse microbiome that supports proper immune function and metabolic health throughout life, with potential long-term consequences for health and disease susceptibility.

Diet and dietary fiber play a crucial role in maintaining gut health and modulating gut microbiome diversity. Indeed, high-fiber diets, particularly those rich in plant-based foods, and prebiotics such as inulin, have been shown to promote a higher diverse microbial ecosystem with an increased abundance of beneficial bacteria such as Bifidobacteria, Roseburia, and Feacalibacterium, known for their anti-inflammatory functions [41,42]. In contrast, Western diets high in processed foods and saturated fats, and low in fiber, have been associated with gut dysbiosis, decreased microbial diversity, and increased intestinal permeability. These dietary patterns have significant effects on the gut microbiome composition and function. For example, studies on indigenous populations, such as the Yanomami in Venezuela and the Hadza of Tanzania, have revealed significantly higher gut microbiome diversity compared to industrialized societies [43,44,45]. Furthermore, plant-based and mediterranean diets promote the production of beneficial bacterial metabolites such as SCFAs while simultaneously lowering the concentrations of trimethylamine N-oxide (TMAO) [46]. SCFAs, particularly butyrate, plays an important role in maintaining gut barrier function and reducing inflammation [47]. Western diets, on the other hand, have been linked to the overgrowth of pathogenic bacteria, a decreased SCFA production, and dysbiosis. These diets can lead to alterations in tight junction proteins, compromising intestinal barrier integrity and allowing translocation of bacterial products such as the LPS into the blood circulation, overall triggering systemic inflammation.

### 1.2. Role of Gut Microbiome in Host Health

One of the primary functions of the gut microbiota is its contribution to host metabolism and nutrition. Gut microbes possess an array of enzymes that allow the breakdown of complex carbohydrates that are otherwise indigestible by the human body. This process, known as fermentation, transforms a large variety of substrates and results in the production of a variety of metabolites such as short-chain fatty acids (SCFAs) including acetate, propionate, and butyrate. These SCFAs serve as an important energy source for colonocytes and have been shown to have anti-inflammatory properties, to contribute to intestinal barrier integrity, and to play a role in regulating host immunity and metabolism [48]. Additionally, certain gut bacteria are capable of synthesizing essential vitamins, including vitamin K and several B vitamins, thus supplementing the host’s nutritional intake [11]. Furthermore, recent research has also highlighted the gut microbiota’s role in xenobiotic metabolism, influencing the processing of various drugs and environmental compounds. The gut microbiome possesses multiple enzymes capable of modifying xenobiotics, potentially altering their bioavailability, functional impact, or toxicity. This microbial metabolic capacity can have significant implications for drug metabolism and personalized medicine approaches [49].

Another crucial function of the gut microbiota is shaping and modulating the host immune system. From early life, the interaction between gut microbes and the host immune system helps to educate and mature immune responses. This process is critical for developing a balanced immune system capable of distinguishing between harmless commensal bacteria and potentially pathogenic microorganisms. The gut microbiota has been shown to influence both innate and adaptive immune responses, affecting the development and function of various immune cell populations [50]. For instance, certain bacterial species have been found to promote the differentiation of regulatory T cells, which are crucial for maintaining immune tolerance and preventing autoimmune diseases [51]. A recent study published in JCI Insight [52] highlights this relationship, focusing on a specific subset of regulatory T cells called DP8α Tregs. These cells, which react to the gut commensal Faecalibacterium prausnitzii, were found to play a protective role against acute graft-versus-host disease (aGvHD) in allogeneic hematopoietic stem cell transplantation patients. The study demonstrated that DP8α Tregs mediate aGvHD prevention in a CD73-dependent manner, likely through host reactivity. This research underscores the intricate relationship between the gut microbiota and immune regulation, suggesting potential therapeutic strategies based on microbiota-induced regulatory T cells.

In addition, disequilibrium in the gut microbiome can initiate a vicious cycle of chronic low-grade inflammation and non-communicable chronic diseases (NCDs). This cycle begins when dysbiosis leads to increased intestinal permeability, allowing bacterial products like lipopolysaccharides (LPSs) to enter the bloodstream. The resulting metabolic endotoxemia triggers a systemic inflammatory response, which in turn further disrupts the gut microbiome composition and function. This disruption exacerbates dysbiosis, leading to a reduced production of beneficial metabolites like short-chain fatty acids (SCFAs) and increased pro-inflammatory cytokines. The persistent inflammatory state creates an environment conducive to the development and progression of NCDs such as obesity, type 2 diabetes, and cardiovascular diseases. These conditions can then further alter the gut microbiome, perpetuating the cycle of dysbiosis, inflammation, and disease [53].

In addition, the microbiota is able to dialog with distant organs, such as the liver, lungs, skin, and even the brain. The gut–brain axis is an area particularly investigated currently. Emerging evidence suggests that the gut microbiota can modulate brain function and behavior through various mechanisms, including the production of neuroactive compounds, stimulation of the vagus nerve, and interaction with the enteric nervous system. This connection has implications for mental health, cognitive function, and neurological disorders [54]. In recent years, SCFAs have been shown to play a significant role in neurodegenerative disorders such as Alzheimer’s disease, Parkinson disease, or amyotrophic lateral sclerosis (ALS). Several studies have demonstrated that the level of butyrate-producing bacteria was lower in patients with these pathologies compared to healthy individuals [7,55]. Interestingly, gut bacteria can produce or stimulate the production of neurotransmitters that regulate gut–brain signaling [56]. Their role in regulating blood–brain barrier permeability has also been highlighted in the literature, affecting which substances can or cannot reach the brain [6]. More investigations are needed to confirm these hypotheses.

**Figure 1:**
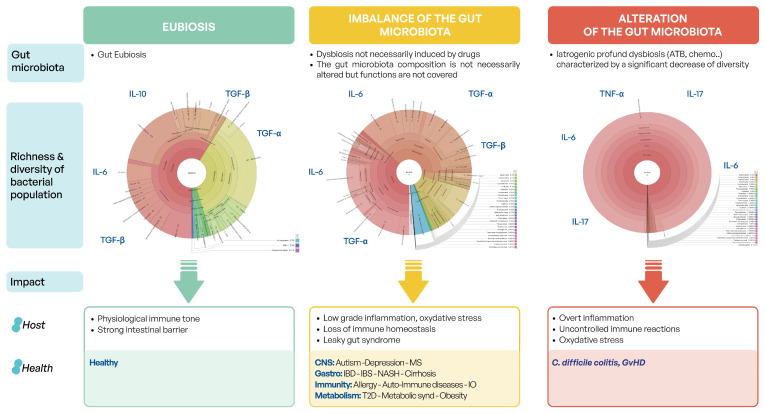
Illustration of the differences between gut microbiome eubiosis, imbalance, and dysbiosis.

Finally, the microbiota serves a function of pathogen colonization prevention via microbe–microbe interactions. As seen in Figure 1, in a healthy gut, flora is finely balanced, and no microbe dominates the ecosystem despite constant attacks by environmental and food bacteria, pathobionts, and other pathogens. This is competitive exclusion. An alteration of gut symbiosis can lead to a loss of resilience and thus, a lasting alteration of symbiosis that is difficult to reverse mostly because dysbiosis is maintained by feedback mechanisms [53]. Hence, it has been demonstrated that dysbiosis directly contributes to severe disorders such as *Clostridioides difficile* infections [57].

## 2. Impact of Dysbiosis in Oncology

The gut microbiome plays a multifaceted and significant role in the development, progression, and treatment of cancers [58]. Indeed, according to a 2021 study [59], 13% of cancer incidence globally has been reported to be caused by microbes. Recent research has highlighted how gut microbial communities can act as both promoters and inhibitors of cancer. Indeed, the microbiome can affect tumor growth directly by producing metabolites that influence cellular pathways or indirectly by modulating the host’s immune response [60,61]. For instance, in some solid tumors, gut microbiome dysbiosis has emerged as a key factor in carcinogenesis.

Altered microbial communities in a dysbiotic state can produce genotoxins and promote or maintain chronic inflammation, potentially leading to DNA damage and cellular transformation [22]. For instance, Escherichia coli strains harboring the pks genomic island produce colibactin, a genotoxin that induces DNA double-strand breaks, chromosomal instability, and increased mutation frequency [62]. Similarly, certain Gram-negative bacteria, including Campylobacter jejuni, Salmonella enterica, and some E. coli strains, produce cytolethal distending toxin (CDT), which causes DNA double-strand breaks and activates DNA damage responses [63,64]. Enterotoxigenic Bacteroides fragilis (ETBF) produces B. fragilis toxin (BFT), which, while not directly genotoxic, induces DNA damage through indirect mechanisms such as activating the Wnt/β-catenin pathway and stimulating inflammatory mediators [65]. Additionally, sulfidogenic bacteria like Desulfovibrio and Fusobacterium species produce hydrogen sulfide (H2S), a genotoxin found in over 80% of sporadic colorectal cancer cases, which directly damages the DNA [66]. These bacteria-derived genotoxins and their associated mechanisms of DNA damage highlight the direct link between specific members of a dysbiotic microbiome and the initiation and progression of tumor formation, underscoring the importance of maintaining a healthy gut microbiome in cancer prevention.

These processes can initiate and accelerate carcinogenesis in various tissues, not limited to the gastrointestinal tract. Furthermore, dysbiosis can disrupt the fine-tuned balance of gut metabolites, which may promote tumor growth and metastasis [67]. For instance, certain bacterial species associated with dysbiosis, such as *H. pylori* in stomach ulcers, can produce metabolites that activate pro-oncogenic signaling pathways. This pathway is classified as a class I carcinogen by the World Health Organization (WHO).

The gut microbiome’s influence on organs beyond the GI tract has specific relevance in cancer. For example, in lung cancer, dysbiosis of the gut microbiota has been associated with altered immune responses and increased susceptibility to carcinogenic factors [68]. Similarly, studies have shown connections between gut dysbiosis and breast cancer, highlighting the far-reaching effects of the microbiome on cancer development [69]

Overall, microbiome has been shown to modulate the tumor microenvironment, influencing critical processes such as angiogenesis, invasion, and apoptosis, which are crucial for solid tumor development and progression [19]. Certain microbial species, or their metabolites, can promote a pro-inflammatory environment that supports tumor growth, while others may have anti-tumor effects. This delicate balance underscores the importance of maintaining a healthy and diverse gut microbiome as a potential strategy for cancer prevention and treatment [70]. In fact, it has been demonstrated that administering specific probiotic bacterial strains (primarily Bifidobacterium and Lactobacillus species) can change the host’s metabolic profile by producing metabolites like serotonin, histamine, γ-aminobutyric acid (GABA), branched-chain amino acids (BCAAs), and short-chain fatty acids (SCFAs) that may be advantageous in specific situations [71]. For instance, in lung cancers, SCFAs have been found to modulate the immune response by promoting the differentiation of regulatory T cells and enhancing the cytotoxic activity of NK cells potentially influencing tumor growth and metastasis [72].

In addition, primary bile acids produced in the liver, which are metabolized by gut bacteria such as Lactobacillus rhamnosus, Bacteroides intestinalis [20], or Clodstrium absonum [73], have been implicated in the development of liver cancer through activation of the G protein-coupled bile acid receptor 1 (GPBAR1) and farnesoid X receptor (FXR), leading to alterations in lipid and glucose metabolisms, inflammation, and cell proliferation [22].

Increased intestinal permeability in a dysbiotic organism can lead to the production of lipopolysaccharide (LPS) by the gut bacteria, which has been shown to trigger a systemic inflammatory response through the activation of pattern recognition receptors like Toll-like receptors (TLRs) on immune cells. This chronic systemic inflammation has been linked to several serious illnesses including chronic ulcerative colitis, and pancreatic and breast cancers [60].

The role of specific microbial species in cancer development and progression is an area of ongoing research. For example, *Fusobacterium nucleatum* has been implicated in colorectal cancer progression, while certain strains of *Escherichia coli* have been associated with an increased risk of colorectal and other cancers [21,74]. Understanding these specific microbe–cancer interactions could lead to targeted interventions for cancer prevention and treatment. Moreover, microbial biofilms, which are structured communities of microorganisms adhering to the surfaces, have been demonstrated to play a significant role in shaping the tumor microenvironment. In colorectal cancer, for example, biofilms composed of bacteria, notably *Fusobacterium Nucleatum* and *Bacteroides Fragilis*, have been found to promote tumor growth by inducing pro-inflammatory cytokines and altering epithelial cell metabolism [75]. These biofilms can also contribute to immune suppression within the tumor microenvironment by promoting the recruitment and activation of myeloid-derived suppressors cells (MDSCs) and regulatory T cells [76].

Additionally, the gut microbiome has been shown to significantly impact the efficacy of various cancer therapies, including radiation therapy, chemotherapy, immunotherapy [68,77,78], and antibiotics. In this context, the microbiome can shape anti-tumor immune responses, diminishing immunotherapies efficacy and patient prognosis [18,79,80]. This interaction between gut microbes and cancer treatments has opened up new avenues for improving therapeutic outcomes. For example, certain gut bacterial species have been associated with improved responses to immune checkpoint inhibitors (ICIs) in melanoma and other solid tumors [23] where the patient response rate is often below 50%. Patients with a more diverse gut microbiome, or those harboring specific bacterial species tend to have better responses to immunotherapy compared to those with less diverse microbiomes or lacking these beneficial bacteria. Generally, species associated with a positive response to ICIs include *Faecalibacterium prausnitzii*, *Bifidobacterium adolescentis, Akkermansia muciniphila*, and members of the *Oscillospiraceae* [Ruminococcaceae] family [81]. Conversely, species like *Bacteroides ovatus* and certain Bacteroidetes have been associated with non-response to ICIs [82]. However, some species like *Ruminococcus bromii* have shown conflicting associations, being linked to both response and non-response in different studies [83,84]. It is important to emphasize that microbial diversity, rather than the presence of specific species, remains the most consistent factor associated with positive ICI outcomes across studies. It is tentative to select a favorable signature to predict immunotherapy response [85,86] since no consensus has been reported on a signature of response in immune-oncological patients [47,51]. Derosa et al. [87] have proposed a signature that could be a predictor of response to ICI that needs to be challenged in clinical trials.

Several clinical trials are ongoing either with single strains (Akkermansia muciniphila [88] or Faecalibacterium prausnitzii [89]), which are associated with better responses to ICIs) or with consortia [90]. So far, approaches using full ecosystem therapies have been the only ones to demonstrate benefit in cancer patients. Furthermore, the microbiome’s influence on chemotherapy efficacy and toxicity is another area of intense research. Some gut bacteria can metabolize chemotherapeutic drugs, potentially altering their efficacy or increasing toxicity [75,91,92]. Understanding these interactions could lead to strategies to enhance treatment efficacy while reducing side effects.

Current research is mainly focused on solid tumors and the role of the gut microbiota in treatment and prevention. Less research is available on the microbiota’s direct impact on treatment responses in liquid cancers. Nonetheless, some differential actions were identified. The microbiota acts differently in hematological cancers and solid tumors. Regarding cancer initiation, specific microbes have been linked to the initiation of certain solid cancers, such as H. pylori in gastric cancer [20]. The role of the microbiota in initiating liquid cancers is less well-established. Intestinal microbes directly interact with the tumor microenvironment, influencing tumor growth and progression [20,93]. In addition, the gut microbiota can regulate the activation of immune cells that migrate to the tumor microenvironment [93], whereas microbiota primarily affects systemic immune responses rather than a localized tumor microenvironment. Regarding colonization, certain bacteria can colonize tumor sites, such as *Bifidobacterium* in mouse models [94], while direct colonization is not relevant for liquid cancers, as they are not confined to specific tissue locations. In the case of tumors, bacterial metabolites in the tumor microenvironment can influence tumor growth and immune responses [93]. A systemic effect is rather observed in liquid cancers.

While the microbiota plays significant roles in both solid tumors and liquid cancers, its effects are more directly observable and extensively studied in solid tumors due to the localized nature of these cancers and their distinct tumor microenvironments.

### Focus on the Role of Gut Microbiome in Hematological Malignancies

The average adult gut microbiome contains over 1000 species-level phylotypes, contributing to its high functional capacity and resilience [30]. As a matter of fact, it has been shown that a higher diversity within the gut microbiota is generally associated with better health outcomes, as it reflects a more stable and robust microbial ecosystem capable of performing a wide range of metabolic and immunological functions [32]. This is particularly demonstrated in patients that are markedly dysbiotic as in hemato-oncology, for example. Indeed, the gut microbiome plays a significant and multifactorial role in hematological malignancies, influencing disease development, progression, and treatment outcomes. Blood cancers account for approximately 6.5% of all cancers worldwide and are characterized by the uncontrolled growth of hematopoietic or lymphoid cells [95]. Recent research has highlighted the complex interactions between the gut microbiota and these blood cancers, revealing that dysbiosis, or an imbalance in the gut microbial composition, can contribute to the pathogenesis and progression of hematological malignancies through various mechanisms [96].

Gut dysbiosis can significantly impact hematological malignancies through several key mechanisms involving the complex interplay between the microbiome and the immune system. The first action is an immune system modulation via the fine tuning of T cell responses and innate immune responses. Certain gut bacteria play a crucial role in shaping T cell responses, which are critical for anti-tumor immunity: segmented filamentous bacteria (SFB) have been shown to induce Th17 cell differentiation [61]. These cells can have both pro- and anti-tumor effects depending on the context. This may suppress excessive inflammation but could also potentially dampen anti-tumor responses. In addition, a mixture of 11 bacterial strains, including seven Bacteroides species, has been identified to induce IFN-γ+ CD8+ T cells both locally in the colon and systemically [61]. In addition, two main mechanisms of innate immune responses were identified. Macrophage polarization triggered by trimethylamine N-oxide (TMAO), a microbiome-derived metabolite, can drive this effect through the NLRP3 inflammasome [50]. This may influence tumor-associated macrophage behavior, and also dendritic cell (DC) modulation through the Syk kinase-coupled signaling pathway, which is critical for the microbiota-induced production of IL-17 and IL-22 by the CD4+ T cell [61]. These cytokines can modulate anti-tumor responses.

The second action is metabolite production. Gut bacteria produce various metabolites that can influence immune function and hematological malignancies such as short-chain fatty acids (SCFAs) and bile acids (BAs). Butyrate is the most studied SCFA. It is produced by fiber-fermenting bacteria and has been shown to enhance the survival and memory responses of activated CD8+ T cells [61]. It also induces extra-thymic Tregs through histone deacetylation inhibition [61]. In addition, certain Clostridiales species enhance the conversion of primary to secondary bile acids, which may affect anti-tumor tolerogenesis.

The relationship between the gut microbiome and hematological cancers is bidirectional. While dysbiosis can contribute to cancer development, the disease itself and its treatments can further alter the microbial composition, creating a vicious cycle. Several mechanisms have been proposed to explain the connection between the gut microbiota and hematological cancers, including epithelial barrier disruption, triggering of local chronic inflammatory responses, antigen dis-sequestration, and molecular mimicry [97,98]. A combination of these mechanisms is likely at play.

Dysbiosis leads to increased intestinal permeability through reduced production of beneficial metabolites like butyrate and overgrowth of pathogenic bacteria. This compromised barrier allows for the translocation of bacterial endotoxins, particularly lipopolysaccharide (LPS), into systemic circulation, a process known as metabolic endotoxemia [99]. In hematological malignancies, where immune dysfunction is often central, this increased endotoxin load activates pattern recognition receptors, triggering the production of pro-inflammatory cytokines such as TNF-α and IL-6. These cytokines play crucial roles in cancer progression: TNF-α enhances tumor invasiveness and promotes malignant cell survival, while IL-6 acts as a growth factor for malignant cells and activates oncogenic signaling pathways [100]. The resulting chronic inflammation creates a tumor-promoting environment, leading to DNA damage, genomic instability, and epigenetic changes that favor cancer initiation and progression. Furthermore, this inflammatory milieu can suppress normal hematopoiesis, promote the expansion of malignant clones, and impair anti-tumor immunity by expanding immunosuppressive cell populations [101]. 

Different hematological malignancies are associated with distinct microbial signatures. For instance, multiple myeloma has been linked to an increased abundance at the species level of *Pseudomonas aeruginosa* and *Clostridium leptum*. These bacteria have been shown to contribute to disease progression through altered metabolism and inflammatory response with the increased activation of inflammatory mediators such as IL-6 and NF-kV pathways, driving myeloma progression [102]. In addition, Prevotella heparinolytica has been linked with an increased intestinal epithelial colonization and bone marrow migration of Th17 cells in myeloma models [102]. While leukemias often show a decrease in Lachnospiraceae, Ruminococcaceae, and *Blautia* [95,103] and an increase in Staphyloccaceae, Streptococcaceae, and *Enterococcus* [104]. The increased presence of Enterococcus in leukemia has been linked with disease progression through immune modulation. Enterococcus faecalis, for example, has been shown to induce the expression of pro-inflammatory cytokines like TNF-alpha by activating p38 MAPK and NF-kB signaling pathways in macrophages, creating a tumor-promoting inflammatory environment [105]. Lymphomas exhibit a moderate reduction in microbiota diversity, with higher proportions of *E. coli* and *C. butyricum* observed in some studies [95]. *E. coli* can produce colibactin, a genotoxin that can contribute to lymphomagenesis through DNA damage [106]. Additionally, studies have shown that patients with Acute Myeloid Leukemia (AML) showed an increased abundance of *Streptococcus*, whereas decreased levels of *Megamonas*, Lachnospiraceae, and *Prevotella* were reported. The increased presence of Streptococcus in AML, as the Streptococcus species has been linked with stimulating the production of IL-6, a cytokine known to promote AML cell proliferation. Moreover, several myeloid leukemia-enriched species were identified, such as *Helicobacter*, *Sphingomonas*, *Lactobacillus*, *Enterococcus*, *Lysobacter*, and *Clostridium* [107]. Regarding multiple myeloma, studies have shown that nitrogen-recycling bacteria, such as *Klebsiella* and *Streptococcus,* were significantly more abundant in patients compared to healthy individuals [102,108]. These microbial composition changes may contribute to MM progression [109]. These alterations in microbial communities can influence the cancer’s development through various mechanisms, including the production of genotoxins, modulation of inflammatory responses, and alteration of metabolite profiles. The microbiome can shape both innate and adaptive immune responses, influencing the development and function of various immune cell populations [110,111]. This interaction between gut microbes and the immune system is particularly relevant in the context of hematological malignancies, where immune dysfunction plays a crucial role in disease progression.

The role of the microbiome becomes particularly crucial in the context of hematopoietic stem cell transplantation (HSCT), a common treatment for many hematological malignancies. The gut microbiota has been shown to significantly impact HSCT outcomes [112,113], including the development of graft-versus-host disease (GvHD), a major complication of allogeneic HSCT. GvHD affects 40% to 50% of patients who have undergone allo-HSCT and has been demonstrated as the leading cause of non-relapse mortality among patients [114,115]. The disease can be subclassified as acute or chronic; acute GvHD (aGvHD) occurs in the first 100 days after allo-HSCT, while chronic GvHD (cGvHD) occurs after those 100 days. In addition, cGvHD affects multiple organs whereas aGvHD symptoms are localized to the skin, liver, and gastrointestinal tract. aGvHD can be graded based on severity from mild (grade I), to very severe (grade IV). The standard first-line treatment for aGvHD is steroids. However, many patients develop a resistance to them and are treated with a second-line treatment, ruxolitinib [116,117]. A considerable proportion of these patients also develop a resistance to ruxolitinib. Management of patients with steroid-resistant and ruxolitinib-refractory aGvHD remains an unmet need.

Numerous studies have consistently shown that aGvHD patients often suffer from gut microbiota dysbiosis, which primarily manifests as a decrease in microbial diversity and an altered composition of specific bacterial taxa [118,119]. This dysbiosis can be attributed to various factors, including the use of broad-spectrum antibiotics, conditioning regimens, and damage to the intestinal epithelium throughout heavy treatment protocols such as therapeutic cycles, and rounds of chemotherapies leading to the transplantation process [120]. Moreover, the complex interplay between antibiotics, the gut microbiome, and GvHD presents a paradox, as antibiotics are often necessary for infection prevention but can also contribute to promoting dysbiosis [121]. This highlights the need for careful consideration of antibiotic use in allo-HSCT patients and the potential for more targeted approaches to microbiome modulation.

Indeed, research has identified several key bacterial groups associated with aGvHD outcomes. As a matter of fact, a decrease in the abundance of beneficial bacteria, including Firmicutes (particularly *Clostridium, Faecalibacterium,* Lachnospiraceae, Ruminococcaceae, Eubacteriaceae, and Peptostreptococcaceae), Bacteroidetes (*Bacteroides* and *Parabacteroides*), and Actinobacteria, has been linked to increased GvHD risk and severity [95]. Conversely, an increase in potentially harmful bacteria, such as Proteobacteria (especially Gamma-Proteobacteria and Enterobacteriales), Verrucomicrobia (*Akkermansia*), and certain Firmicutes (Staphylococcaceae, and *Enterococcus*), has been associated with worse aGvHD outcomes [29,122,123].

Mechanisms by which the gut microbiota influences aGvHD development are multifactorial. Microbial metabolites play a significant role in modulating the immune response and maintaining intestinal homeostasis. For instance, short-chain fatty acids (SCFAs) produced by certain bacteria have anti-inflammatory properties and can help in maintaining intestinal barrier integrity because intestinal epithelial cells use them as an important energy source supporting their function and survival [124]. Butyrate and propionate play a crucial role in influencing immune responses and maintaining gut barrier integrity in the context of graft-versus-host disease (GvHD). They both promote the differentiation of regulatory T cells, which are critical for maintaining immune tolerance [125]. In addition, butyrate has been found to enhance the repair of damaged intestinal epithelial cells, which is particularly important in the context of GvHD-induced gut damage [125]. In addition, lower hexanoate and elevated isobutyrate concentrations were also observed in patients who developed cGvHD [125]. SCFAs serve as an important energy source for intestinal epithelial cells, supporting their function and survival [124]. The loss of SCFA-producing bacteria during allo-HSCT may contribute to increased inflammation and more pronounced GvHD severity [48,126].

Additionally, it has been demonstrated that gut microbiota signals indirectly affect regulatory T cells (Tregs) by activating CD103+ CD11b+ dendritic cells. These dendritic cells then promote Treg anti-inflammatory responses through TGF-β and retinoic acid production. Tregs maintain immune tolerance and aid tissue repair by producing amphiregulin, which is crucial for gut flora homeostasis and immune modulation, especially in the context of GvHD [125,126,127,128]. Furthermore, the translocation of bacteria and microbial products across a damaged intestinal barrier can trigger inflammatory responses and activate donor T cells, exacerbating GvHD. The interaction between microbial antigens and pattern recognition receptors on host cells can lead to the recruitment of inflammatory cells involved in GvHD pathogenesis [129].

A study by Burgos da Silva and colleagues [130] explored microbiome changes that may have organ-specific effects in GvHD. They found that the composition and metabolism of the microbiome before GvHD onset affect organ involvement in GvHD and are predictive of GvHD-related patient outcomes. This work expands earlier ideas that different pathogenetic processes contribute to organ-specific GvHD. Additionally, this study revealed more severe dysbiosis, with specific alterations of the microbiome, as well as major changes in the bacterial metabolic activity in lower gastrointestinal tract GvHD (LGI-GVHD). Indeed, they showed that patients with a lower abundance of members of the Clostridia class, mostly *Blautia* species in the peri-engraftment period, were associated with an increase in GvHD-related mortality and overall survival.

Another recent study has also highlighted the importance of the pre-transplant microbiome in influencing GvHD onset. A study by Koyama et al. [131] published in *Immunity* used animal models of GvHD and complex computational analyses to identify bacteria that help instigate GvHD, and others that suppress it. Their work showed that the pre-transplant microbiome could be the focus of modulation to reduce GvHD severity. The study found that certain bacterial genera appeared to induce MHC class II (MHCII) expression, while others suppressed it. MHCII expression on intestinal epithelial cells plays a crucial role in the development of GvHD. Mice with higher levels of gut MHCII were found to develop more severe GvHD.

Hence, understanding the complex interactions between the gut microbiome and GvHD has led to the exploration of microbiome-based interventions for GvHD prevention and treatment. Strategies such as fecal microbiota transfer (FMT), probiotic supplementation, and targeted antibiotic use have shown promises in restoring microbial diversity and potentially reducing GvHD risk [132].

## 3. Microbiome-Based Approaches to Improve Patients’ Lives

Given the significant impact of the microbiome on hematological malignancies and treatment outcomes, there is a growing interest in microbiome-modulating strategies as adjunct therapies. These approaches include the use of probiotics, prebiotics, synbiotics, microbes consortia, and full ecosystems to restore a healthy gut microbiome and potentially improve treatment outcomes [133,134].

### 3.1. Prebiotics, Probiotics, and Synbiotics

The use of prebiotics, non-digestible food ingredients that selectively stimulate the growth and activity of beneficial gut bacteria, could positively influence the microbiota of HSCT recipients. Indeed, Yoshifuji and colleagues [135] conducted a prospective study on 30 HSCT patients given a prebiotic formula containing glutamine, fiber, and oligosaccharides, along with resistant starch-rich meals. Compared to a 142-patient control group, the prebiotic group experienced less severe oral mucositis and diarrhea. Notably, 17% of prebiotic patients avoided diarrhea entirely versus 7% of controls. The study also observed a 10% lower incidence of GI aGvHD in the prebiotic group. Hence, evidence suggests that dietary choices significantly influence HSCT outcomes by modulating the gut microbiota. However, additional research is necessary to fully understand this relationship.

Mechanisms by which prebiotics modulate immune pathways involved in GvHD prevention are multiple. Prebiotics, such as fructo-oligosaccharides (FOS) and galacto-oligosaccharides (GOS), selectively promote the growth of beneficial bacteria like Bifidobacteria and Lactobacilli. These bacteria produce short-chain fatty acids (SCFAs), particularly butyrate. Butyrate has been shown to enhance the differentiation of regulatory T cells (Tregs) through histone deacetylase inhibition, thereby promoting immune tolerance [136]. Additionally, SCFAs can directly interact with G protein-coupled receptors on immune cells, modulating their function and reducing inflammatory responses [122].

Probiotics, which can be a single strain or a consortium of specific, identified and characterized live microorganisms, when administered in adequate amounts, have been shown to potentially confer health benefits to the host, particularly in GvHD management. Researchers have investigated various probiotic strains for their potential to reduce GvHD severity and improve outcomes. For example, a study by Gerbitz and colleagues [137] demonstrated that administration of *Lactocaseibacillus rhamnosus* GG reduced GvHD-related mortality in a mouse model, by enhancing intestinal barrier function and modulating T cell responses. More recently, Gorshein et al. [138] reported that a probiotic mixture containing *Lactobacillus* and *Bifidobacterium* strains reduced the incidence of acute GvHD in allo-HSCT patients. The mechanisms of action for these probiotics are strain-specific and diversified. Bifidobacterium longum, for instance, has demonstrated the ability to reduce pro-inflammatory cytokine production and enhance Treg differentiation, potentially mitigating GvHD severity [139]. Escherichia coli Nissle 1917 has been found to compete with pathogenic bacteria for intestinal colonization and modulate dendritic cell function, potentially reducing the risk of infections and GvHD [140]. These probiotic strains contribute to immune homeostasis through various mechanisms, including the production of antimicrobial substances, modulation of cytokine production, enhancement of intestinal barrier function, and modulation of T cell responses [139]. However, more research is needed to determine the most effective probiotic strains and optimal dosing regimens for GvHD prevention and treatment with more assuring data on its safety.

Synbiotics comprise a combination of probiotics and prebiotics. Yazdandoust and colleagues [141] conducted a study examining the effects of a synbiotic supplementation on transplant outcomes. In their research, 40 patients were divided into two equal groups: one receiving daily synbiotic capsules containing various probiotic strains (including *Lactobacillus*, *Bifidobacterium*, and *Streptococcus* strains), and a control group receiving no intervention. Results were promising, with the synbiotic group experiencing no bloodstream infections and a significantly lower incidence of aGvHD by day 100 post-transplant (10% vs. 40% in the control group). The study found that synbiotic intake before and during the conditioning regimen led to a reduction in the incidence and severity of acute GvHD through the induction of CD4+CD25+Foxp3+ regulatory T cells. While larger studies are needed to confirm these findings, they suggest that carefully designed microbial consortia could have significant potential in improving HSCT outcomes. These findings suggest that synbiotic intake may reduce GVHD incidence. The metabolites produced by beneficial bacteria, particularly SCFAs, contribute significantly to immune homeostasis in the context of transplant-related complications. Butyrate, for example, enhances epithelial barrier function, induces the differentiation of colonic Tregs, suppresses the activation of nuclear factor-κB in intestinal epithelial cells, and modulates the function of antigen-presenting cells, potentially reducing the activation of alloreactive T cells. These mechanisms collectively contribute to the potential benefits of synbiotics in HSCT recipients.

### 3.2. Microbes Consortia

This approach consists of providing consortia of isolated and characterized strains of microorganisms aimed at restoring key functions missing in patients. The selection of microbial strains for these consortia is based on their specific metabolic and immunomodulatory roles, with a focus on restoring functions critical for immune homeostasis and reducing inflammation. In the context of GVHD, while few developments were reported, the principles applied in other conditions can be relevant. For example, the selection of strains often prioritizes those capable of producing short-chain fatty acids (SCFAs), particularly butyrate, which has been shown to play a crucial role in maintaining epithelial barrier integrity and modulating immune responses [142]. Few developments have been reported in GvHD, whereas in GI tract disease and in the treatment of solid tumors, several companies reached clinical phases, such as Nubiyota (Guelph, ON, Canada) with its recently published results for a phase II/III (NCT03686202) [143] in HPV-related locoregionally advanced oropharyngeal cancer squamous cell carcinoma or Vedenta with its ongoing phase III for the prevention of recurrent *Clostridioides Difficile* infection (NCT06237452).

Vedanta’s (Cambridge, MA, USA) VE303 consortium has demonstrated significant efficacy in preventing recurrent C. difficile infection. In their phase II CONSORTIUM trial, treatment with VE303 was associated with a 30.5% adjusted absolute risk reduction in the rate of recurrence when compared with placebo, representing a greater than 80% reduction in the odds of a CDI recurrence [144]. Furthermore, VE303 accelerated the restoration of a healthy gut microbiome community and early recovery of key metabolites. Among nearly 400 bacterial species detected in the study participants after treatment, species in VE303 were the top predictors of non-recurrence. These results highlight the potential of rationally designed bacterial consortia in addressing complex microbial dysbiosis-related conditions.

The ROMA2 study investigated the use of microbial ecosystem therapeutic 4 (MET4), an oral consortium of cultured human stool-derived immune-responsiveness associated bacteria, in combination with chemoradiation for patients with HPV-related locoregionally advanced oropharyngeal cancer squamous cell carcinoma (LA-OPSCC). This phase II trial aimed to evaluate the safety and efficacy of gut microbiome modulation in boosting anti-tumor immune responses. Twenty-nine patients received at least one dose of MET4, with drug-related adverse events occurring in 13 patients, mostly grade 1–2. While the study did not meet its primary ecological endpoint of increased MET4 relative abundance in stool samples post-intervention for the overall cohort, exploratory findings suggested potential engraftment in stage III patients. This subgroup showed higher MET4 relative abundance at week 4 and 2-month follow-up, correlating with changes in plasma and stool metabolomics. These results warrant further investigation of microbiome interventions, particularly in stage III LA-OPSCC patients undergoing primary chemoradiation [98].

The emphasis on the consortia’s ability to target specific pathophysiological conditions is rooted in the understanding that multiple strains can provide complementary functions. For instance, some strains may be selected for their ability to produce SCFAs, while others may be chosen for their capacity to enhance mucus production or modulate specific immune cell populations. In designing these consortia, companies consider several factors: metabolic complementarity, immune modulation, colonization resistance, and stability and engraftment. Strains are often selected to have complementary metabolic functions, creating a network of interdependent organisms that can stably colonize the gut. Different strains may target various aspects of the immune system, such as promoting regulatory T cell development or modulating dendritic cell function. The ability of the consortium to prevent colonization by pathogenic organisms is a key consideration, especially in conditions like C. difficile infection. Strains are also chosen for their ability to persist in the gut environment and effectively colonize the intestinal mucosa [145].

### 3.3. Full Ecosystem Approach

#### FMT

Fecal microbiota transfer has emerged as a promising approach for both preventing and treating GvHD. FMT involves transferring fecal microorganisms from a donor to a recipient for restoring microbial diversity and beneficial bacterial populations, and thereby a functional host-microbes symbiosis. FMT preparations are typically produced and used in hospitals and clinics by academics. They are sourced from healthy donors, with occasional use of relatives or partners as donors in selected cases, but also the patient himself or a patient who survived a disease. Administration methods are diverse, including rectal enemas, nasoduodenal or nasojejunal tube infusions, colonoscopies, and oral capsules. FMT preparations are generally derived from a single donor, and thus composition can be highly variable from one preparation to another [146]. Over the years, the potential of combining multi-donor FMTs have been highlighted to mitigate the risk of selecting ineffective or non-compatible donors [147] and to increase the richness and diversity of the gut microbiome [148], which could improve the efficacy of FMT [149]. By restoring a diverse and rich gut microbiome, FMT is aimed at restoring immune homeostasis and competitive exclusion potential, thereby potentially reducing GvHD incidence and severity.

The primary therapeutic target for FMT in these studies was steroid-resistant (SR) or steroid-dependent (SD) gastrointestinal GI aGvHD. Common adverse events associated with FMT are primarily gastrointestinal, such as abdominal discomfort, bloating, and diarrhea. A major concern in FMT administration is the risk of infectious complications. To mitigate this risk, FMT is typically performed after the resolution of aplasia and in the absence of significant GI symptoms or toxicity. This approach is aimed at reducing the likelihood of bacterial infections during the neutropenic phase and limit bacterial translocation through a compromised intestinal barrier. To maintain the diversity and richness of the transferred microbiota, antibiotic therapy is typically discontinued at least 48 h before FMT and withheld for 48 h post-procedure.

Mechanisms by which FMT exerts its beneficial effects in GvHD are complex and diverse. Researchers have proposed several potential mechanisms, including the following:

1. Restoration of microbial diversity: FMT can help to re-establish a diverse and balanced gut microbiome close to the one of a healthy person. It exerts competitive exclusion towards potential pathogens and is associated with improved GvHD outcomes [150].
2. Production of beneficial metabolites: Restored microbial communities can produce SCFAs and other metabolites that have anti-inflammatory properties and help maintain intestinal barrier integrity [48].
3. Modulation of the immune response: Studies have shown that the gut microbiome can influence the balance between regulatory T cells and pro-inflammatory Th17 cells, which may impact GvHD severity [122].
4. Restoration of intestinal barrier function: FMT may help repair damage to the intestinal epithelium caused by conditioning regimens and GvHD itself, reducing bacterial translocation and inflammation [151].
5. Competitive exclusion of pathogens: A healthy, diverse microbiome can help prevent the overgrowth of potentially harmful bacteria that may exacerbate GvHD; in fact, they found that patients with low microbial diversity had significantly lower 3-year overall survival (36%) compared to those with high diversity (67%) [123].

In recent years, several clinical trials have demonstrated the potential efficacy of FMT in GvHD management. For instance, a randomized controlled trial (NCT02269150) conducted by Taur and colleagues [152] investigated the effects of autologous FMT in HSCT recipients. Their preliminary results showed successful restoration of microbiota diversity and composition close to pre-transplant profiles in patients receiving autologous FMT. The intervention effectively recovered commensal groups such as Lachnospiraceae, Ruminococcaceae, and other Bacteroidetes. Goeser and colleagues described in 2021 [153] two German center case series (*n* = 11) of FMT administered via a nasojejunal tube or oral capsules as a rescue therapy for GI SR-GvHD. Six patients were also on ruxolitinib at the time of FMT. Pre- and post-FMT microbiota investigation demonstrated lower α-diversity before FMT, but elevated after FMT, though never reaching donor levels. The β-diversity analysis revealed that pre-FMT, post-FMT, and donor microbiota samples grouped separately, with a shift in post-FMT sample composition towards donors’ profiles. The microbiota composition revealed that FMT increased Ruminococcaceae, Bacteroidaceae, Lachnospiraceae, Streptococcaceae, and Lactobacillaceae, while decreasing Akkermansiaceae, Enterococcaceae, Veionellaceae, Peptostreptococcaceae, and Clostridiaceae. Seventeen patients who were subjected to HSCT and received FMT by nasoduodenal infusion for SR or SD grade II–IV GI aGvHD were enrolled by Van Lier and colleagues in a clinical study [154]. Gastrointestinal problems were the most common adverse event recorded and disappeared within a few hours. A total of 50% SR GI aGvHD and 78% SD GI aGvHD showed a full GvHD response. Reaction to FMT was translated into a reduced GvHD grade during therapy. Six of the patients who experienced a complete response continued to respond following the cessation of immunosuppression, whereas four individuals displayed a secondary failure. GvHD complications claimed the lives of four out of the five non-responding patients.

In another non-randomized, open-label, phase I/II trial (NCT03148743) with 41 patients suffering from SR GI aGvHD, Zhao and affiliates [155] observed that by day 14, 52.2% of the 23 patients that received a nasojejunal FMT reached clinical remission, with an overall response rate of 82.6%, whereas the control group’s was only 39%. Overall, FMT restored a higher microbial diversity in recipients, including a higher abundance of Proteobacteria and a decreased prevalence of Firmicutes. Bacteroidetes were more commonly found in stool samples from patients with SR GI aGvHD. After FMT, Firmicutes abundance increased while Proteobacteria dropped. A subset cohort of the prior trial, comprising 21 patients with grade III–IV SR GI aGvHD treated with FMT and ruxolitinib, was described by Liu and his team [156]. After a median of 10 days, 10 complete responses were observed, and the overall response rate was 71.4%. Eighty percent of respondents presented sustained improvement. In one third of instances, there was a GvHD relapse. Reactivations of viruses (62%), bacterial infections (29%), and severe cytopenia (81%), were the most common adverse events. Responders showed a decrease in activated T cells, inflammatory cytokines like interleukin IL-2 and IL-17A, and an increase in Tregs. Additionally, respondents showed a decrease in *Escherichia* and an increase in *Lactobacillus*.

Rashidi and its team [157] conducted a randomized, phase II, placebo-controlled trial (NCT03678493) evaluating oral FMT capsules in HSCT recipients. The study reported a higher incidence of GvHD in the FMT arm (18.4% vs. 0% in placebo), but a lower infection density (0.74 vs. 0.91 per 100 patient/days). Microbiota analysis revealed increased α-diversity and enrichment of the Coriobacteriaceae and Rikenellaceae families post-FMT, while reducing *Enterococcus* and oral bacteria like *Dialister*. In 2024, they completed the study with a multi-omics analysis from the sample of the trials and found that post-FMT expansion of *Faecalibacterium*, associated with donor microbiota engraftment, predicted a higher risk for aGVHD. Under homeostatic conditions, the commensal genus *Faecalibacterium* has gut-protective and anti-inflammatory properties. Nonetheless, they indicated that it may become harmful in the context of FMT following allo-HSCT [119]. More recently, DeFilipp and colleagues [158] conducted a single-center, open-label phase II trial investigating the use of third-party FMT for treating high-risk, treatment-naïve aGVHD of the lower gastrointestinal tract. The study enrolled 15 patients who received 2–3 FMT doses via enema over 7 days, alongside standard GVHD prophylaxis. Results were promising, with an overall response rate of 80% at day 28 and 73% at day 90. Complete response rates were 47% at day 28 and 60% at day 90. The 6-month overall survival rate was 80%, with a 12-month rate of 77%. GVHD-free, failure-free survival at 6 months was 60%. No serious adverse events were attributed to FMT. Microbiome analysis showed increased α-diversity and enrichment of beneficial bacteria post-FMT. While these findings suggest that third-party FMT is a promising treatment for this condition, the authors emphasize the need for larger, randomized studies to confirm these results and potentially establish FMT as a standard treatment option.

Overall, efficacy of FMT in treating SR/SD GI aGvHD varied considerably across studies, with response rates ranging from 28% to 75%. This wide range underscores the heterogeneity of outcomes and the need for further research to optimize FMT protocols in the context of HSCT and GvHD management [154,159,160]. Moreover, as for any other modality of treatment, robust clinical data will only be obtained with high quality products. Additionally, these clinical trials were conducted by academics involving a limited sample of patients and little standardization requirements. As a result, this not only restricts the number of patients treated but also limits the geographic reach and the potential for pharmaceutical-grade application.

### 3.4. Standardized Donor-Derived Products

To maximize patient outcome reproducibility, industrial players have implemented standardized manufacturing processes to develop drug candidates using fecal material as a source material. These compositions are sourced from strictly vetted donors according to the GMP manufacturing process, allowing standardization of the products and safety for the patients. Different modalities exist, using either single-donor or pooled-donor-based compositions. When using pooled products, for example, for MaaT013 produced by Maat Pharma for the treatment of GvHD, batches are manufactured by pooling fecal material from three to eight strictly vetted, healthy donors. The safety testing strategy comprises medical evaluation, and regular testing of blood and feces following current regulatory recommendations for safety testing. Each manufacturing campaign includes the qualification of healthy donors, daily stool collection, batch manufacturing, close donor health follow-up, and product quality control, leading to batch release. Each stool from a participating donor is mixed independently from the others with a cryopreservative diluent to allow optimal preservation conditions. The pooled suspension is then distributed into 150 mL freeze-resistant bags and stored at −80 °C. Pooling allows the standardization of the product composition and intra-batch consistency regarding the relative abundance of the main phyla including specific genera associated with clinical benefits, such as butyrate-producing bacterial genera.

**Figure 2:**
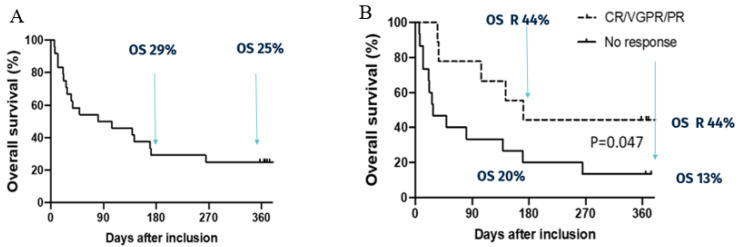
Patients’ response and outcomes after MaaT013 treatment in the HERACLES study based on Malard et al. [161] (**A**) Overall survival in HERACLES (**B**) Overall survival according to response to MaaT013 in HERACLES.

In the case of aGvHD, the most advanced development is a pooled allogenic donor-derived product, called MaaT013, which is currently tested in a phase III study (see Table 1). Results from the phase II of this product (NCT03359980) were published by Malard and colleagues [161]. This study was a single-arm phase IIa on 24 patients with SR GI aGvHD and 52 patients from the expanded access program (EAP). All patients received at least two doses of MaaT013, a pooled allogenic donor-derived product comprising a high species richness and high microbial diversity, administered via enema. The authors demonstrated that MaaT013 exhibited lower batch variability and higher microbial richness as compared to single donors. As seen on Figure 2, at day 28 (D28), a 38% GI-overall response rate (ORR) in the prospective population was observed, comprising five complete responses (CR), two very good partial responses (VGPR), and two partial responses (PR). The GI-ORR was 58% (seventeen CR, nine VGPR, and four PR) in the EAP. In the prospective research, the 12-month overall survival (OS) was 25%, whereas in the EAP, it was 38%. 

In terms of safety, it was not possible to rule out the possibility that three of the five infectious complications—including sepsis—were connected to the trial protocol. According to investigations using shotgun sequencing, none of the infectious strains that were discovered were present in MaaT013. Overall, the microbiota composition analysis revealed an increased richness and alpha diversity after treatment at any time point. 

Several active clinical trials assessing microbiome-based therapies as prophylaxis or as adjuvant therapy for SR aGvHD are conducted, as reported in Table 1. The most advanced in the field of microbiome-based therapy for SR GI aGvHD is a phase III (NCT04769895) multicenter open-label trial to evaluate the efficacy of MaaT013 after their successful phase II. Additionally, Dougè et al. [162] initiated a multicenter, randomized, phase II clinical trial (NCT04935684) to evaluate the impact of FMT on 1-year GvHD-free relapse-free survival in patients undergoing myeloablative HSCT. This ongoing study aims to provide further insights into the efficacy of FMT in modulating HSCT-related morbidity and mortality. While most studies have focused on donor-derived microbiome-based therapies as a treatment for established GvHD, there is growing interest in its preventive potential with an increasing number of active clinical trials. For instance, three clinical-stage biotech companies are conducting trials using standardized microbiome-based therapies. Mat Pharma is currently the only biotech company investigating standardized microbiome-based drugs for both the treatment and prevention of aGvHD and it is also the company with the most advanced pipeline in these indications.

**Figure 3:**
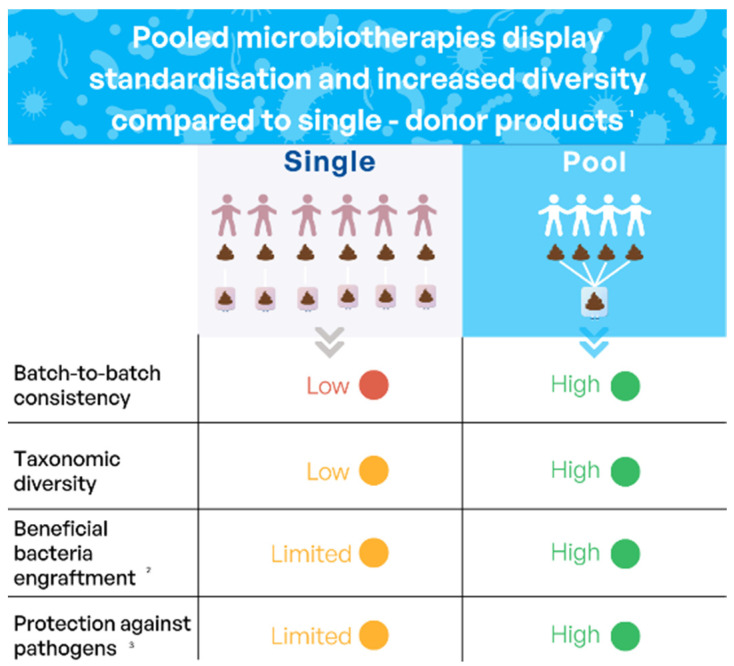
Pooled versus mono-donor approaches in mouse model based on Reygner et al. [99]. ^1^ Infectious murine models; ^2^ *C. difficile* infectious murine model; ^3^ Two pathogens tested on murine models (*S. enterica* serotype Typhimurium and *C. difficile*) and three pathogens tested on growth inhibition assay (*C. difficile*, *E. faecium* vana and *K. pneumoniae* oxa48).

Microbiome-based therapies, especially pooled standardized donor-derived products, appear to restore microbial diversity, promote the engraftment of beneficial bacterial species, and modulate the immune response, potentially leading to improved clinical outcomes. Indeed, a recent study by Reygner et al. [146] showed, has illustrated in Figure 3, that the pooling strategy for these microbiome-based therapies demonstrated a more homogeneous, diverse, and enriched product, compared to individual donors. Additionally, it demonstrated that while the effectiveness of individual donors varied, pooled products decreased the pathogenicity of *Salmonella* and *C. difficile* in mice. The antimicrobial potential of pooled microbiome-based therapies was proven by in vitro assays against *Klebsiella pneumoniae* oxa48 (KP) and *Enterococcus faecium* vanA (EF).

Numerous studies have consistently shown that patients with hematological malignancies and those undergoing allogeneic hematopoietic stem cell transplantation (allo-HSCT) often suffer from gut microbiota dysbiosis, characterized by a decreased microbial diversity and an altered composition of specific bacterial taxa. This dysbiosis can contribute to the development and progression of hematological cancers, as well as increase the risk and severity of GvHD in allo-HSCT recipients. The mechanisms by which gut dysbiosis impacts these conditions are multifaceted, including modulation of the immune response, production of pro-inflammatory metabolites, and disruption of the intestinal barrier function. In addition, recent advances in high-throughput sequencing technologies and bioinformatics have revolutionized our understanding of the gut microbiome’s composition and diversity, revealing a collective microbial genome that exceeds human genome by a factor of 150 [163]. Diversity within the gut microbiome is typically measured using alpha diversity metrics, which account for both species’ richness (the number of different species) and evenness (the relative abundance distribution of these species) [164]. This genomic complexity underscores the profound impact of the microbiome on human biology and highlights the need for continued research to elucidate its multifaceted roles. Moreover, the integration of microbiome analysis into standard risk assessment and treatment planning for allo-HSCT patients is likely to become more common, allowing for more tailored approaches to patient care.

## 4. Conclusions and Perspectives

In conclusion, the growing body of evidence supporting the crucial role of the gut microbiome, particularly gut dysbiosis, in hematological malignancies and GvHD, opens new avenues for therapeutic interventions. While challenges remain due to the complexity of the gut microbiome and the need for more randomized clinical trials and standardized procedures, the potential for microbiome-based therapies to complement existing treatments and improve patient outcomes represents an exciting frontier in hematology–oncology research and clinical practice. Continued efforts towards the standardization of gut microbiome analysis methods, including stool collection preservation, DNA extraction, and bioinformatic analysis, are essential to ensure the reliability and clinical applicability of microbiome research. This standardization will allow for interlaboratory comparison of results and alignment on signatures and features correlated to disease. More randomized controlled trials are needed to provide robust and convincing data on the therapeutic role of microbiota as a new treatment modality, enabling long-term safety studies and exploration of the long-term effects of microbiome modulation on immune function and cancer recurrence [165]. Further investigation of these long-term effects on cancer recurrence and overall survival in hematological malignancy patients will be crucial for establishing the full potential of these approaches. The exploration of engineered bacteria to deliver targeted therapies or modulate immune responses in the context of hematological cancers and GvHD also holds promise for developing novel therapeutic strategies. As our understanding of the microbiome’s role in these conditions continues to grow, we can anticipate the development of more targeted and personalized microbiome-based interventions, including co-cultivated full ecosystem approaches, engineered bacteria, or rationally designed bacterial consortia for specific patient populations. These advancements bring us closer to a future where personalized, microbiome-informed approaches become an integral part of cancer care and the management of transplant-related complications.

## Figures and Tables

**Table 1:** Identified clinical trials testing microbiome-based therapies for GvHD treatment and prophylaxis. According to the Company’s presentation and website, Enterobiotix has initiated a phase IIa for a pooled standardized donor-derived product aimed at preventing post allo-HSCT complications, with the first patient expected to be dosed in H1 2024.

Trial n°	Sponsor	Start/End Date	Status	Phase	Enrollment	Microbiome-Based Approach	Summary
	*Microbiome-based approaches as treatment*						
*NCT04769895*	MaaT Pharma (Lyon, France)	25/03/2022 30/09/2024	Recruiting	Phase III	75	Pooled standardized donor-derived product	Multicenter open- label trial to evaluate the efficacy of MaaT013 as salvage therapy in patients with aGvHD refractory to ruxolitinib
*NCT03819803*	Medical University of Graz	01/03/2017 31/12/2026	Recruiting	Phase III	15	FMT	Endoscopically FMT for patients with aGvHD
*NCT04269850*	St. Petersburg State Pavlov Medical University	01/09/2019 01/12/2025	Recruiting	Phase I/II	20	FMT	Pilot study of FMT in combination with ruxolitinib and steroids to treat saGvHD
*NCT05067595*	Fred Hutchinson Cancer Center	17/10/2024 (estimated) 31/12/2026	Recruiting	Phase I	72	FMT	Study to evaluate the role of FMT and a dietary fiber supplementation in treating GvHD
*NCT03148743*	The First Affiliated Hospital of Soochow University	16/05/2017 12/2024	Recruiting	Obs.	50	FMT	Study to evaluate the safety and efficacy of FMT for gut GvHD
	*Microbiome-based approaches as prophylaxis*						
*NCT05762211*	MaaT Pharma (Lyon, France)	06/11/2023 15/02/2027	Recruiting	Phase IIb	387	Pooled standardized donor-derived product	Randomized placebo-controlled double-blind to evaluate the protective effect of MaaT033
*NCT06026371*	Fred Hutchinson Cancer Center	12/12/2023 09/30/2026	Recruiting	Phase II	138	FMT	Randomized placebo-controlled double-blind trial to test whether FMT prevents aGvHD during HCT
*NCT04935684*	University Hospital, Clermont- Ferrand	20/12/2022 12/2027	Recruiting	Phase II	150	FMT	Randomized placebo-controlled double-blind trial to assess FMT efficacy in the prevention of post allo-HSCT complications, particularly GvHD
*NCT04373057*	Duke University	22/01/2021 01/02/2028	Recruiting	Phase I/II	128	Prebiotics	Determine whether carbohydrate prebiotics can modulate the microbiome and help prevent GvHD
*NCT04995653*	Seres Therapeutics, Inc. (Cambridge, MA, USA)	24/11/2021 10/2024	Completed	Phase Ib	60	Microbe consortium	Open-label, randomized placebo-control double-blind, multicenter study to evaluate the safety, tolerability, pharmacokinetics, and efficacy of SER-155 to prevent aGvHD
*NCT04745221*	The First Affiliated Hospital of Soochow University	01/03/2021 01/03/2026	Recruiting	N/A	100	FMT	Study to evaluate the safety and efficacy of autologous FMT in preventing aGvHD

## Data Availability

No new data were created or analyzed in this study. Data sharing is not applicable to this article.
